# Mechanobiomaterials: Harnessing mechanobiology principles for tissue repair and regeneration

**DOI:** 10.1016/j.mbm.2024.100079

**Published:** 2024-05-16

**Authors:** Xiao Lin, Hua Yang, Yi Xia, Kang Wu, Fengcheng Chu, Huan Zhou, Huajian Gao, Lei Yang

**Affiliations:** a Orthopedic Institute, Department of Orthopedics, The First Affiliated Hospital, Soochow University, Suzhou, China; b School of Mechanical Engineering, Hebei University of Technology, Tianjin, China; c Center for Health Sciences and Engineering (CHSE), Hebei Key Laboratory of Biomaterials and Smart Theranostics, School of Health Sciences and Biomedical Engineering, Hebei University of Technology, Tianjin, China; d Mechano-X Institute, Applied Mechanics Laboratory, Department of Engineering Mechanics, Tsinghua University, Beijing, China

**Keywords:** Biomaterials, Mechanics, Mechanobiomaterials, Tissue repair and regeneration, Mechanobiology

## Abstract

Mechanical stimuli are known to play critical roles in mediating tissue repair and regeneration. Recently, this knowledge has led to a paradigm shift toward proactive programming of biological functionalities of biomaterials by leveraging mechanics–geometry–biofunction relationships, which are beginning to shape the newly emerging field of mechanobiomaterials. To profile this emerging field, this article aims to elucidate the fundamental principles in modulating biological responses with material–tissue mechanical interactions, illustrate recent findings on the relationships between material properties and biological responses, discuss the importance of mathematical/physical models and numerical simulations in optimizing material properties and geometry, and outline design strategies for mechanobiomaterials and their potential for tissue repair and regeneration. Given that the field of mechanobiomaterials is still in its infancy, this article also discusses open questions and challenges that need to be addressed.

## 1 Introduction

The repair and functional restoration of tissue lesions (e.g., cartilage defects, intervertebral disc degeneration, volumetric muscle loss, and myocardial infarction) remain a great challenge for clinicians, biologists, and biomedical engineers. Compared to chemical drugs and other therapeutic agents, various implantable and wearable biomedical devices have already been playing equal, if not more important, roles in mediating tissue repair and functional restoration. The physiochemical characteristics of biomedical devices, especially non-active devices, are largely rooted in their constituent materials and subsequently affect the biological responses of the human body. To date, the influence of the biochemical cues of materials on cellular responses has been widely investigated, providing a foundation for the rational design of a material's chemical characteristics for tissue repair and regeneration. In addition to biochemical cues, the mechanical interaction between biomedical devices and tissues is ubiquitous. However, traditional mechanical factors have only been considered for the development of load-bearing biomedical devices to guarantee their reliability in the body's loading environment.

From a mechanics perspective, human and animal tissues have specialized structures and mechanical properties that enable an appropriate response or tolerance to dynamic loadings during physiological activities. In addition, the extracellular matrix (ECM), with varying composition and architecture, transduces mechanical forces at the tissue level to the mechanical stimuli experienced by cells. To accommodate the dynamic mechanical environment, cells sense extracellular mechanical stimuli and regulate their metabolic activities accordingly.^1^ However, tissue damage or degeneration can dramatically change the mechanical stimuli perceived by cells. Trauma- or aging-induced pathological changes in tissue mechanical (including matrix degradation, loss of tissue hydration, and production of incorrect fibrous ECM) and structural properties (including changes in tissue shape, size, and microarchitecture) result in substantial changes in stress and strain distribution across tissues. At the cellular/subcellular scale, the degradation of the ECM affects the mechanical interaction between the ECM and cells and consequently alters the mechanical stimuli experienced by cells in a particular zone. Therefore, traumatic damage and/or pathological changes in tissues at different length scales can result in abnormal mechanical stress and strain, even under normal physiological loads, causing dysregulation of mechanobiological signaling of cells, compromising tissue reparative capacity, and/or even damaging adjacent healthy tissues. For example, an altered subchondral bone structure after osteoarthritis (OA) aberrantly changes the patterns of mechanical stress within the articular cartilage (AC), which subsequently disturbs chondrocyte metabolism and AC homeostasis, causing degeneration of the AC and continuous progression of OA pathology.^2^

Correction of the aberrant cellular mechanical environment and rebuilding tissue mechano-adaptive processes can potentially prevent or reverse tissue pathogenesis. Meanwhile, a number of important mechanobiological signaling pathways and molecular mechanisms related to tissue regeneration have been identified,^3^^,^^4^ allowing new strategies to boost tissue regeneration and repair.^5^^,^^6^ Recently, an improved understanding of cellular mechanobiological behavior and its critical role in mediating tissue growth and homeostasis^3^^,^^7^^,^^8^ has led to advances in the rational design and development of biomaterials to regulate the responses of the surrounding tissue/cells through mechanical interactions.^9^ Over the past decade, many studies have reported enhanced tissue repair and regeneration by modulating the mechanical properties and geometry of biomaterials.^10^, ^11^, ^12^

Mechanobiomaterials is an emerging field of research in which biomaterials are proactively designed or programed with target mechanical properties, responses or behaviors for precisely mediating biomechanical environment of living systems, in order to regenerate or restore partially or totally any tissue, organ or to function of the body. The major objective of mechanobiomaterials is to design biomaterials that can dynamically and consistently adapt and respond to the changing mechanical environment of tissues and cells to enhance their regenerative potential through appropriate mechanical stimuli.^9^^,^^13^ This is a fast-emerging field at the interface of biology, biomechanics, materials science, and engineering, aimed at developing mechanically responsive or active materials that can interact with cells or tissues and thus proactively alter or program their mechanobiological responses. A large amount of research in this field has focused on modulating or programming cell behavior (e.g., proliferation, migration, protein secretion, and differentiation) via biomaterial-induced mechanical cues to enhance tissue repair and restoration. Modulating the mechanical properties and geometries of materials has already demonstrated great potential for repairing heart,^10^ intervertebral disc,^11^ cartilage,^14^ muscle^15^ and skin^16^ injuries as well as opening up new applications such as skin appearance improvement,^17^ orodental reconstruction,^18^ and intestinal circumferential expansion.^19^

Numerous studies have investigated development strategies for mechanobiomaterials and their underlying biological and biomechanical mechanisms. The present article provides an overview of this emerging area of research, summarizing the fundamentals of material–tissue mechanical interactions at multiple scales, the relationships between material mechanics and biological responses, and the principles or strategies for designing mechanobiomaterials for tissue repair and regeneration. Hopefully, this study will foster future research in this area and bolster the evolution of next-generation biomaterials and biomedical devices.

## 2 Biomechanical and mechanobiological insights into biomaterial design

Correction of pathological or stimulation of reparative mechanobiological pathways through the regulation of cellular mechanical environment at lesion sites could enhance tissue regeneration during the clinical treatment of tissue lesions. Interactions between biomaterials and tissues/cells occur at multiple length scales, and wearable and implantable materials can deliver mechanical stimuli (e.g., stretch, compression, and shear) to cells either in contact with biomaterials or within tissues, eliciting biological responses at the molecular, cellular, and tissue levels through mechanobiological mechanisms.^20^, ^21^, ^22^ Compared with chemical or biological stimuli, mechanical stimuli provide a relatively predictable platform for the precise control and modulation of cellular responses. Understanding how biomaterials induce mechanical stimuli and how biological systems respond to such stimuli are the two pillars for the rational design of mechanobiomaterials.

### 2.1 Mechanical interactions between biomaterials and tissues/cells

**Fig. 1:**
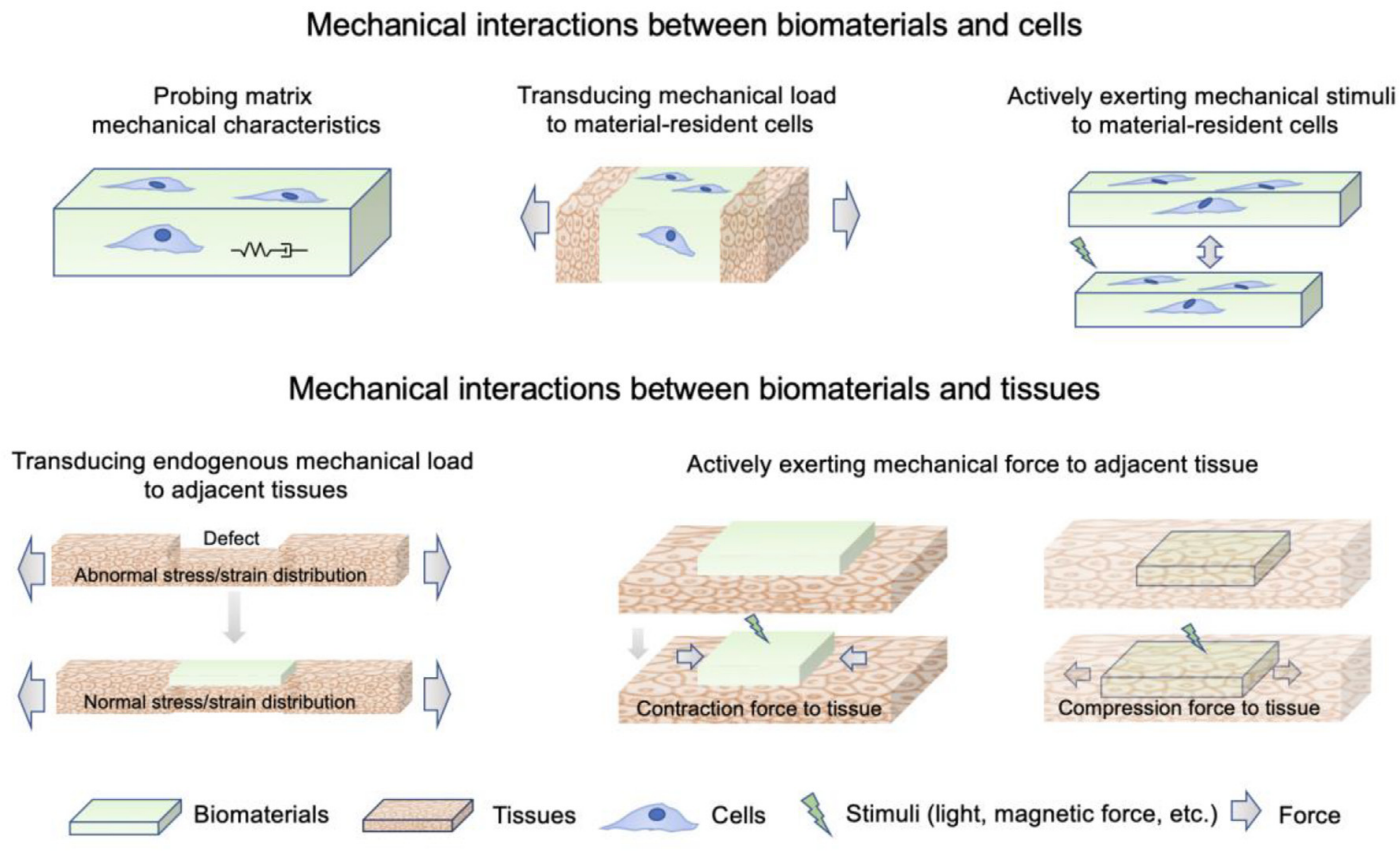
Mechanisms involved in mechanical interactions between biomaterials and tissues/cells.

The mechanical interplay between biomaterials and tissues/cells involves multi-scale interactions (Fig. 1). At the cellular or subcellular scale, living cells constantly push or pull the surrounding ECM. When in contact with cells, biomaterials can trigger mechanobiological responses through cell-probing mechanisms, and their mechanical properties can affect the mechanical stimuli perceived by cells and subsequently dictate cellular function and fate.^21^ It should be mentioned that the topographical features of biomaterials play a crucial role in governing cellular behaviors by affecting the mechanical interplay between cells and biomaterials.^23^^,^^24^ At the tissue or organ scale, endogenous mechanical forces are constantly present throughout the tissue or organ in various forms (e.g., heartbeat, lung expansion, and joint/muscle compression). The presence of biomaterials may change the dynamic mechanical environment of the tissue (e.g., dynamic distribution of stress and strain), and thus alter the mechanical stimuli perceived by cells. For example, the stress and strain distributions within biomaterials and local tissues can be significantly altered, depending on the mechanical properties and geometries of the biomaterials. In addition, responsive biomaterials that change their mechanical properties and geometry under external stimuli (e.g., electric or magnetic force, vibration, and light) can directly exert mechanical stimuli on tissues and cells.

The mechanical environment of tissue-resident endogenous cells can be regulated based on an understanding of material–tissue mechanical interactions at multiple scales. Given that exogenous cells are usually incorporated into biomaterials for applications, such as cell-based therapy, mechanical stimuli to these cells can also be controlled by material–tissue mechanical interactions. Mechanical stimuli can be perceived by cells and can affect cell fate through various mechanisms, as discussed in the next section.

### 2.2 Transduction of mechanical signals at cellular level

**Fig. 2:**
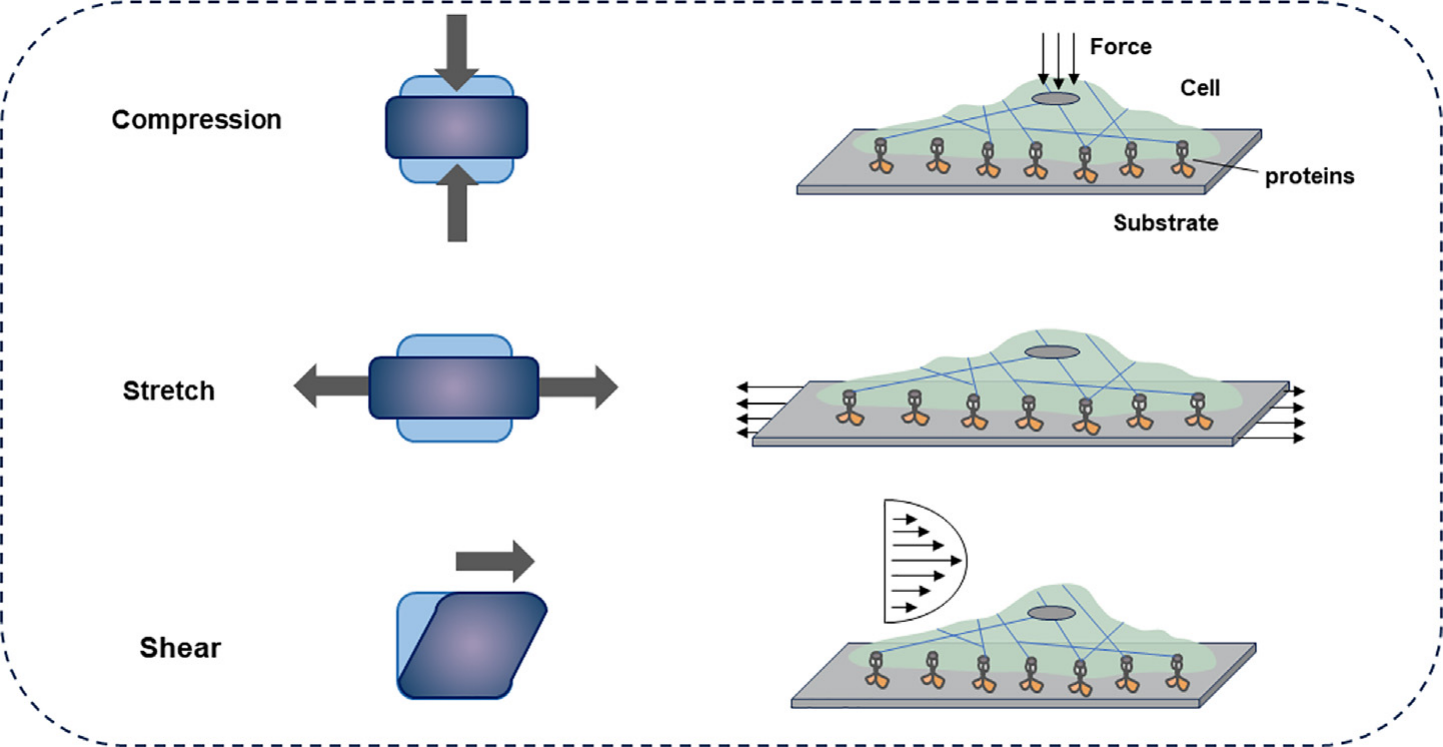
Typical modes of mechanical stimuli experienced by cells. External forces result in compressive, stretch (tensile), and shear stresses on cells.^26^

Cells perceive and respond to extracellular and intracellular mechanical stimuli, which is crucial for tissue development, homeostasis, and regeneration.^25^ Extracellular mechanical stimuli (such as stretching, compression, and tissue shearing) (Fig. 2)^26^ and intracellular stimuli (such as osmotic pressure and cytoskeletal forces generated by the cytoskeleton interacting with the plasma membrane and intracellular organelles)^27^^,^^28^ can arise in static, incremental, and/or cyclical forms. Cells within tissues are subject to a variety of forces, to which they can either adapt or react by altering their shapes. Experimental setups can be devised to replicate these conditions and investigate how cells react to applied forces under controlled circumstances. Among the most prevalent types of physical forces exerted on living cells are pressure, stretching, and fluid shear. The specific types of forces experienced by cells vary depending on the methods and substrates used to apply the forces.^29^

Cell stretching devices typically utilize elastic membranes as substrate materials. These devices stretch cells adhering to the membrane either by directly stretching the elastic basement membrane itself or by inducing deformation through methods such as liquid or vacuum. Cell stretch devices concentrate on studying the impacts of stretch direction, frequency, intensity, and duration. Various stretching modes such as uniaxial, biaxial, and equiaxial can be applied to cells within these devices. Cell compression system is designed to exert mechanical pressure on living cells. For example, the uniform compression method involves placing a glass cylinder with uniform and steady compression on a slab of hydrogel or plate over confluent cell layers in large quantities.^30^ Shear stress could be exerted through a microfluidic device capable of incrementally adjusting magnitude across substrates with varying stiffness levels.^31^ The mechanical characterization of cells necessitates measurement techniques capable of precisely applying and monitoring deformations at the micro-to nanometer scale and forces in the nano-to piconewton range, given the cells' small size and low elastic modulus. Additionally, approaches that assess local cellular mechanical properties could exert extracellular dynamic forces in the nano-to piconewton range across the entire cell, known as force-application techniques, including optical tweezers (laser traps), atomic force microscopy (AFM), acoustic force spectroscopy (AFS), cell stretchers, flow rheometry and so on.^32^

Mechanobiological transduction is the ability of living cells to respond to numerous mechanical stimuli and convert them into intracellular signals to direct cell behaviors (cell movement, secretion, endocytosis, proliferation, differentiation, apoptosis, etc.).^33^^,^^34^ Tension transduction is a typical mechanism involved in cell perception and transduction of mechanical stimuli, and is mainly transduced by the cell membrane and actin cytoskeleton. Another typical mechanism is mediated by mechanosensitive ion channels, which can be mechanically activated to allow ions to selectively traverse the cell membrane.^35^

**Fig. 3:**
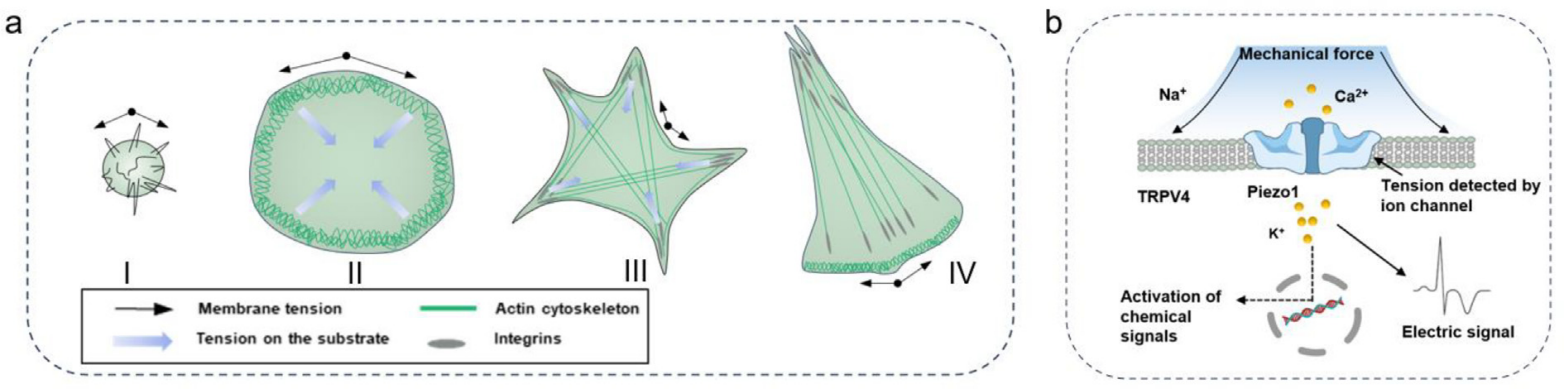
Typical mechanisms involved in cell reception of mechanical signals. (a) Model of cell spreading and polarization. Phase 1 involves rounded up cells with weak cell–substrate attachment but higher membrane tension. In phase 2, the cells begin to spread by unfolding membrane reservoirs and increase the membrane tension. Stable adhesions start to mature during phase 3, global remodeling of the cytoskeleton occurs with formation of actin bundles and the membrane tension is now at its low resting level with a constant membrane area. In the final polarized phase, lamellipodia protrude on one side of the cell, whereas the other side of the cell shrinks and membrane tension increases.^38^^,^^59^, ^60^ (b) Schematic diagram of ion channel activation by mechanical force. Schematic diagram of Piezo1 channel activation by mechanical force.^61^

Tensile stress mainly includes membrane tension due to osmotic pressure differences across the lipid bilayer and cytoskeleton tension originating from actin attached to the plasma membrane.^36^ Cells control membrane tension by modulating membrane area and cytoskeletal attachment,^37^ as shown by the membrane area regulation/cytoskeleton model of cell spreading and polarization.^38^ Fig. 3(a) shows typical phases during the cell spreading and polarization processes. Rounded cells exhibit weak cell–substrate attachment and high membrane tension. Stable adhesion and global remodeling of the cytoskeleton decrease membrane tension. The protrusion and shrinking of lamellipodia on opposite sides of the cell increase the membrane tension in the polarized phase.

In the basal equilibrium stress state, integrins are in equilibrium with the actin filaments (F-actin) and ECM. Mechanical stimulation of cells alters the stress equilibrium instantaneously, leading to remodeling of the cytoskeleton, which in turn influences the transduction of forces throughout the cell. For example, cells polymerize cytoskeletal proteins to create stress cables and induce actomyosin contraction or self-loading to match applied loads.^39^^,^^40^ Variations in cytoskeleton length, density, alignment, and cross-linking, together with their kinetics and dynamics, directly regulate the mechanical properties of cells, such as contractility, rigidity, motility, and various cellular responses.^41^, ^42^, ^43^ Meanwhile, there is a dose-dependent relationship between the degree of externally applied force and cytoskeletal stiffness, and the interconnected structural tensile elements globally re-orientate in response to local stress in a manner consistent with the tensegrity model.^44^^,^^45^

Mechanosensitive ion channels are force sensors that translate mechanical inputs into electrical and/or biochemical intracellular signals.^46^ When mechanical stimulation reaches the cell membrane, stress is distributed to all components, including the double layer, cytoskeleton, and ECM, which pulls the ion channels and transforms them from a closed state to an open state (Fig. 3(b)), allowing specific ions to flow based on channel selectivity.^47^, ^48^, ^49^, ^50^ Mechanosensitive ion channels are known to sense various mechanical signals, including radial pressure, membrane stretching, compression, shear stress, matrix stiffness, ultrasound, matrix nano-topology, and osmotic pressure,^51^^,^^52^ and their opening probability increases with the strength of mechanical stimuli.^53^ The activation of ion channels by different mechanical stimuli mediates diverse cell and tissue functions^46^^,^^54^^,^^55^ and pathological processes.^56^, ^57^, ^58^

In addition to the aforementioned mechanisms, cells can detect mechanical stimuli via growth factor- and primary cilia-mediated mechanisms.^62^ Mechanical stimuli induce changes in mechanical receptors on the cell membrane and result in a biochemical cascade, often referred to as a signaling pathway,^63^ leading to the modulation of gene expression in the nucleus. Focal adhesions (FAs) with mechanosensitive qualities are able to convey mechanical forces between the extracellular matrix and the cytoskeletal contractile machinery.^64^ Mature FAs comprise multiple molecular layers. The layer adjacent to the membrane is known as the integrin signaling layer, housing essential FA signaling proteins like focal adhesion kinase (FAK) and its adapter protein paxillin.^65^

**Fig. 4:**
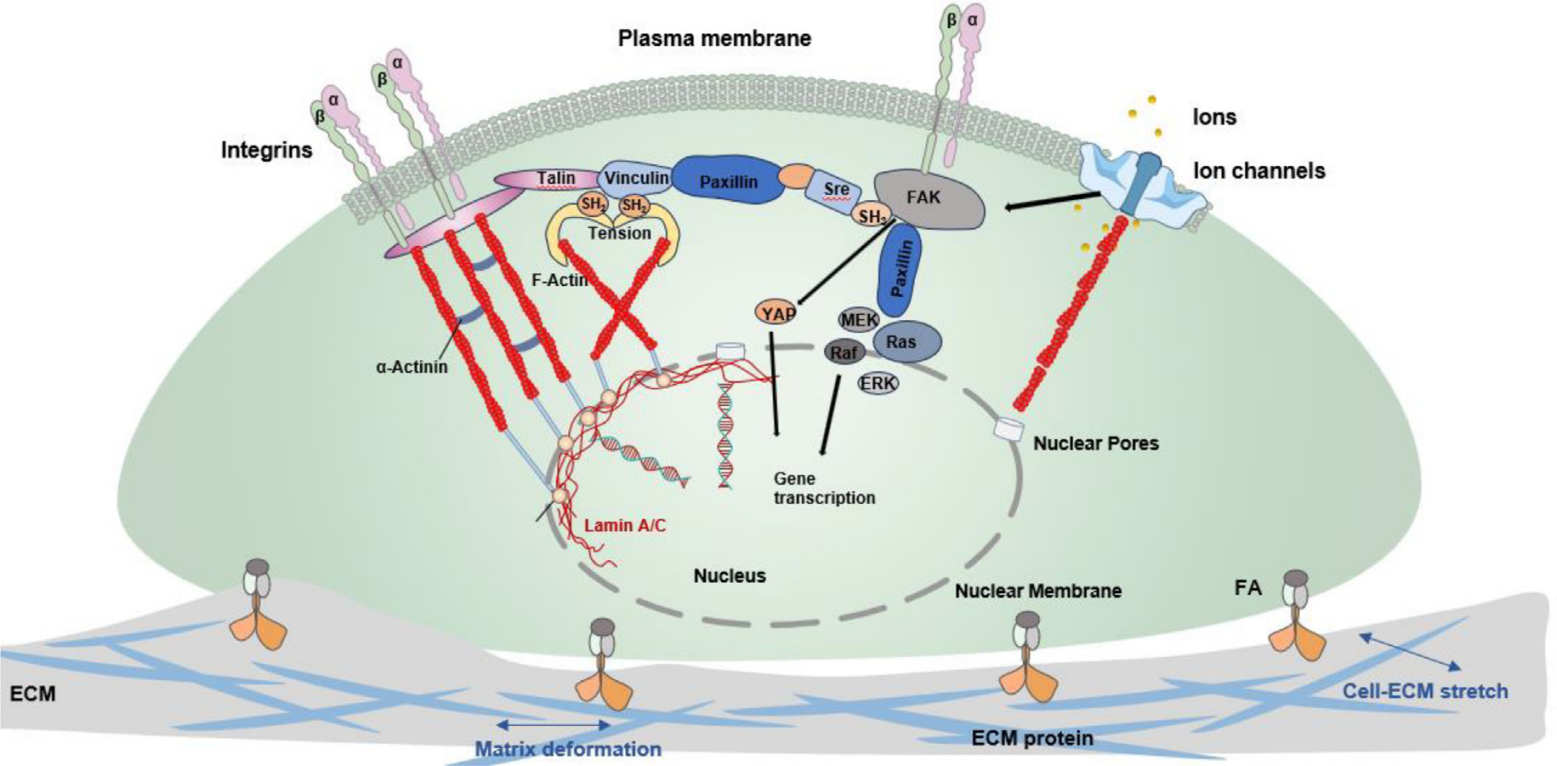
Representative schematic showing several typical mechanisms involved in cell mechanotransduction.

As shown in Fig. 4, FAK pathway is a model of many avenues through which mechanical signals can be used to regulate adaptive responses.^66^ FAK can be activated by either integrins or growth factors, and tyrosine phosphorylation of FAK can trigger the assembly and disassembly of focal adhesions and cadherin-based ECM.^67^ FAK assumes a pivotal role in modulating the FA assembly and disassembly, thereby governing the directional mobility of cells.^68^ Activation of FAK undergoes autophosphorylation of Tyr-397 (FAK Y397), creating a binding site for the Src homology 2 (SH2) domain of Src and related kinases. This initiates a cascade where Src phosphorylates FAK at numerous other tyrosine residues, serving as docking points for various signaling molecules like Grb2, p130cas, and phosphatidylinositol 3-kinase, ultimately activating extracellular signal-regulated kinases (Erks). Rigid substrates foster heightened and prolonged integrin clustering, with increased clustering density enhancing FAK phosphorylation in a linear fashion. This augmented integrin clustering density prolongs FAK Y397 phosphorylation by facilitating FAK re-binding to integrin via talin, thereby extending the reaction duration.^69^ Mechanical activation by the compression of Piezo channels induces calcium influx, which stimulates downstream signaling pathways, such as Src and extracellular signal-regulated protein kinase (ERK), finally leading to the formation of actin-based protrusions. Shear flow, substrate stiffness, and enlarged focal adhesions lead to YAP activation in the cytoplasm and its translocation to the nucleus, where YAP triggers gene transcription, resulting in increased cell proliferation and other functions.^70^^,^^71^ Various mechanosensors and signaling pathways may play synergistic roles in ensuring a robust mechanosignalling program.

The mechano-adaptive changes in morphology of cells can result in the physical extension or contraction of nuclear pores, influencing the transport of molecules between the cytoplasm and nucleus, and subsequently modulating gene expression levels.^72^^,^^73^ Microtubules are recognized as essential regulators of nuclear morphology, which in turn modulates transcriptional activity through chromatin condensation and the expression of downstream genes involved in cellular mechanics.^74^

**Fig. 5:**
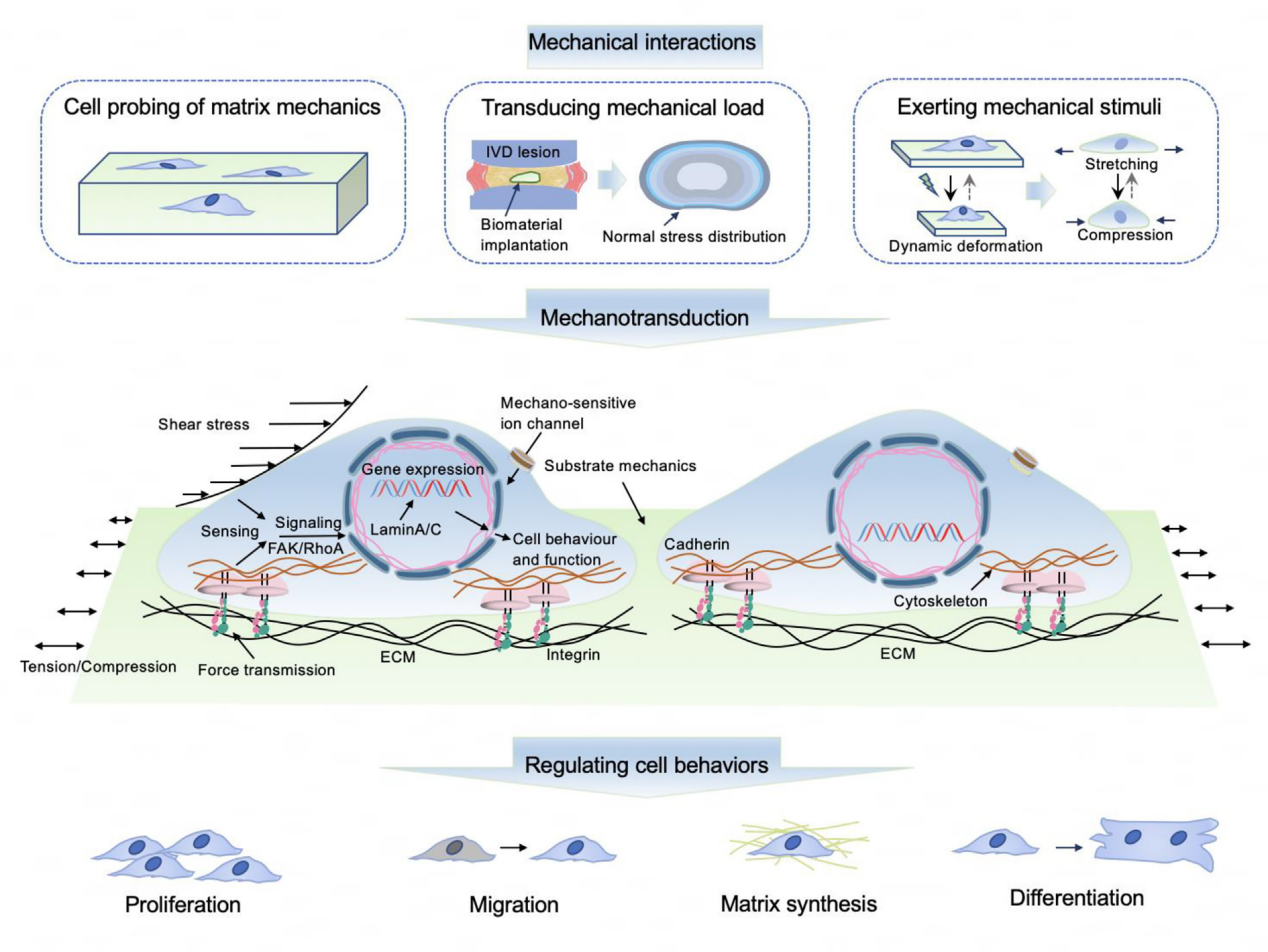
Illustration of typical mechanotransduction mechanisms steering material- and tissue-resident cell behaviors. IVD: intervertebral disc.

This biomechanical and mechanobiological understanding suggests that by optimizing the mechanical properties of biomaterials, it is possible to construct an appropriate mechanical microenvironment for tissue-resident or exogenous cells to regulate their behavior, prevent pathological remodeling, and promote tissue regeneration (Fig. 5). Numerous studies have demonstrated that the mechanical properties of biomaterials and their (stimuli-responsive) programming strategies regulate the mechanical signals perceived by cells that adhere to the biomaterials or within local tissues. The effects of intrinsic and (stimuli-responsive) dynamic mechanical properties on cellular and tissue responses are discussed in the following section to better illustrate the concept and rational design of mechanobiomaterials.

## 3 Effect of material mechanics on cellular and tissue responses

Numerous studies have revealed the influence of extracellular mechanics on the phenotype and function of various cells (Table 1). In addition, the mechanical properties of implants have been found to regulate adjacent tissue responses.

**Table 1:** Cellular processes that are potentially affected by matrix mechanical properties.

Stiffness		

Matrix	Biological processes	Ref.

High-density collagen-I gels	Maintenance, proliferation, stratification, and survival of limbal epithelial stem cells	105
Polyethylene glycol hydrogel	Stemness and proliferative efficiency of skeletal muscle stem cell	107
Alginate-RGD hydrogels	Matrix remodelling of bone marrow mesenchymal stem cells	106
Hybrid gold nanoparticle-hyaluronic acid hydrogel	Electrical and contractile performance of human induced pluripotent stem cells	108
Trilayer extracellular matrix-based microribbon scaffold	Deposition of cartilage-like collagen and synthesis of sGAG of human mesenchymal stem cells	109
Beta-sheet rich silk nanofiber and amorphous silk nanofiber composite hydrogel	Chondrogenic-osteogenic gradient differentiation of bone mesenchymal stem cells	189
Proteolytically degradable alginate hydrogels	Migration and invasion of human mesenchymal stem cells	111
Alginate microgel	*In vivo* residence time of clonally derived mouse marrow stromal cells after intravenous injection	112
Four-armed poly(ethylene glycol) hydrogel	Organoids generation of human pluripotent stem cell	116
Gelatin-hydroxyphenylpropionic acid hydrogel	sGAG production and gene expression of chondrocyte	103
γ-PGA-SH/OHA-GMA hydrogel	Migration and infiltration of fibroblasts	92
Hyaluronic acid-based microrods	Reprograming of myofibroblast	95
Alginate hydrogel	Osteogenic differentiation of mesenchymal stem cells	110
Biphasic CAN-PAC hydrogel	Viability of cartilage cells and osteoblast cells	96
**Viscoelasticity/dynamic network**		
**Matrix**	**Biological processes**	**Ref.**
Hyaluronate–alginate hybrid hydrogel	Chondrogenic differentiation of mouse chondrocytes (ATDC5 cells)	190
Benzimidazole-based catalyst enhanced hyaluronic acid hydrogel	Long-term survival and adhesion of human umbilical vein endothelial cells (HUVEC)	148
Supramolecular gelatin hydrogels	Spreading, tension of cytoskeletal and osteogenesis of human mesenchymal stem cells	147
PEG-gellan gum/PEGDA double network hydrogel	Proliferation, spreading and chondrogenic differentiation of bone mesenchymal stem cells	157
DEX-UPy dynamic hydrogel	Osteogenic differentiation of bone mesenchymal stem cells and chondrogenic expression of chondrocyte	155
Peptide supragel	Embedding and spheroid harvesting of cancer cells (MCF-7 and 4T1)	161
PPy-based dynamic conductive hydrogel	Differentiation towards astrocytes of neural stem cells	164
Gelatin methacryloyl –fibrinogen interpenetrating network hydrogel	3D myoblast alignment and elongation of C2C12 cells	165
Collagen, alginate, and PEDOT:PSS biohybrid hydrogel	Physiological beating rate of human-induced pluripotent stem cell–derived cardiomyocytes	166
3D hydrogel structure based on reversible receptor–ligand interaction between the glycopeptide antibiotic vancomycin and dipeptide D-Ala-D-Ala	Adhesion and morphology of L929 cells and HUVECs	167
Alginate hydrogel	Osteogenic differentiation and migration of hMSCs	169
GelMA hydrogels	Spreading, proliferation, osteogenesis and chondrogenesis of bone mesenchymal stem cells	14
PVA/Glycerol hydrogel	Vitality of nucleus pulposus cells	11
**Strain-stiffening effect**		
**Matrix**	**Biological processes**	**Ref.**
Polyisocyanides gel	KCa3.1 channel expression of HepG2 cells	130
**Viscosity**		
**Matrix**	**Biological processes**	**Ref.**
Polydimethylsiloxane-based substrate	Collective movement of epithelial cells	181
Hyaluronic acid hydrogel	Cell spreading of human bone marrow-derived mscs	182
Poly(ε-caprolactone)-based substrate	Spread area and formation of spheroids of NIH/3T3 fibroblast	183
Supported lipid bilayers	Differentiation of C2C12 mouse myoblasts	184
Gelatin solution	Adipogenic and osteogenic of human bone marrow–derived mesenchymal stem cells	186
	ECM production and expression of collagen type II/aggrecan of chondrocytes	185
	Chondrogenic differentiation of human mesenchymal stem cells	187
High-viscosity storage solution (EAS-1587)	Storage of red blood cell and inflammatory cytokines expression of human mesenchymal stem cells	188

Numerous mathematical or physical models have been developed to understand and describe the mechanical interactions between biomaterials and biological systems at the tissue, cellular, and subcellular levels, as discussed in several review papers.^75^^,^^76^ These models have helped understand the role of mechanical cues in tissue degeneration for the rational design of mechanobiomaterials.

**Fig. 6:**
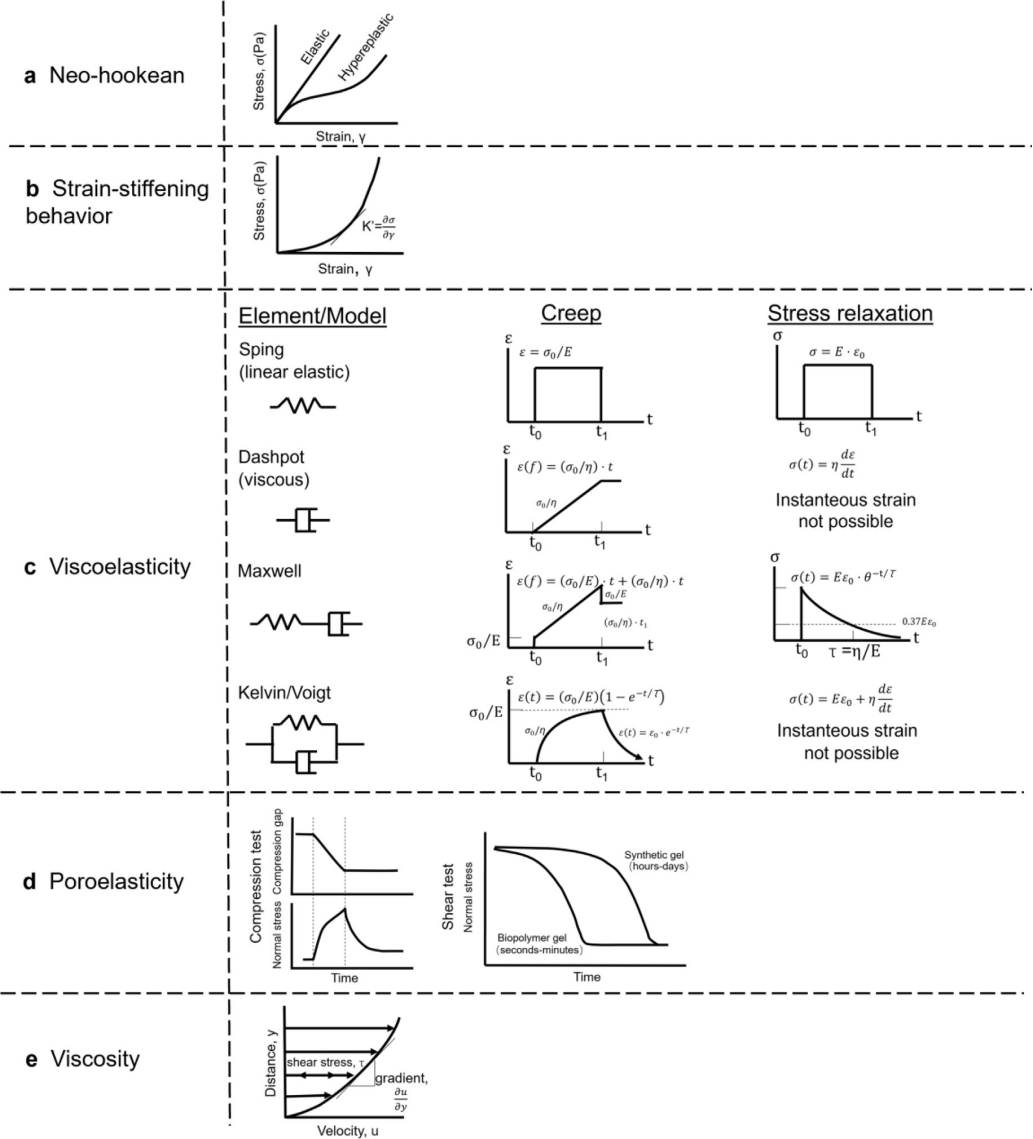
Schematics of typical constitutive models for describing mechanical properties of biomaterials. (a) The stress–strain curve for a hyperelastic (Neo-Hookean) model. (b) The stress–strain curve of the polyisocyanopeptide hydrogel in a stress ramp. (c) Viscoelastic creep and stress relaxation under an instantaneous and constant applied stress (σ_0_) and strain (ε_0_).^136^ (d) Poroelastic behaviors of biopolymer networks under compression and shear tests.^173^ (e) Newton's law of viscosity.

Among various mechanical properties of materials (Fig. 6), some are related to rate- or time-independent responses to load (not changing with time or strain rate, including stiffness, strain-stiffening behavior, etc.), while others elicit time-dependent responses (changing with time or strain rate, including viscoelasticity, poroelasticity, viscosity, etc.).^35^^,^^77^, ^78^, ^79^, ^80^, ^81^ In this section, the effects of these mechanical properties on cell and tissue responses are summarized, and their biological implications are discussed.

### 3.1 Time-independent mechanical properties

#### 3.1.1 Stiffness/elasticity

When a constant stress or strain is applied to elastic materials, they respond with a strain or stress that also remains constant over time, storing mechanical energy until the force or deformation is released, while the material fully returns to its original configuration. Stiffness is a key parameter describing the response of an elastic material to a load. With a linear elastic isotropic model, the stiffness is usually defined by Young's modulus =σε , where σ=F/A, ε=ΔL/L, σ is stress, ε is strain, F is force, A is area, and ΔL is change in length. Constitutive modelling of biomaterial stiffness can be challenging because both the nonlinear and anisotropic nature must be properly handled, whereas a linear elastic isotropic model is not always valid. Often, a hyperelastic isotropic incompressible material with a neo-Hookean material model is used to describe the mechanical behavior of biomaterials.^82^ Usually, the strain energy density of a neo-Hookean material is defined as

$$W=C_{10}\left(I_{1}-3\right)$$

where $I_{1}$ is the first deviatoric strain invariant and $C_{10}$ is a material parameter. A stress–strain curve of a neo-Hookean model material is schematically presented in Fig. 6(a). Models of more complex biomaterials that consider anisotropic properties are increasingly being developed.^83^, ^84^, ^85^, ^86^, ^87^, ^88^

Cell behaviors, including cell spreading, migration, ECM deposition, proliferation, stem cell lineage commitment, and self-renewal of a variety of cell types are affected by the stiffness of the substrate or material to which they attach (Table 1).^78^, ^79^, ^80^ Here, the effects of biomaterial stiffness on immune responses, the efficacy of cell therapy, and the activity of tissue-forming cells are briefly discussed for guiding biomaterial design.

Biomaterial stiffness influences the behavior of endogenous cells.^89^ First, the stiffness of the substrate determines cell migration and tissue integration in synthetic scaffolds.^90^, ^91^, ^92^, ^93^, ^94^ Secondly, substrate stiffness affects the phenotypic reprogramming of endogenous cells.^95^^,^^96^ Third, the inflammatory responses of innate immune cells, which play a central role in regulating tissue injury repair, are also influenced by the stiffness of the biomaterial, as seen in *in vitro*^97^^,^^98^ and *in vivo* studies.^93^^,^^99^ However, the correlation between matrix stiffness and immune responses remains to be fully elucidated as mixed results have been reported. Either increased^98^^,^^100^ or decreased^101^^,^^102^ pro-inflammatory macrophage phenotypes with increasing substrate stiffness have been observed in different studies.

Biomaterial stiffness affects the efficacy of cell therapy.^91^^,^^103^ In particular, biomaterial stiffness modulates the regenerative capacity of stem cells.^104^ First, the substrate stiffness affects the maintenance, activity, and proliferation of stem cells.^105^, ^106^, ^107^, ^108^ The specific lineage commitment of stem cells has been found to be controlled by substrate stiffness.^109^^,^^110^ Substrate stiffness significantly affects cell migration from the scaffold to the host tissue. For example, softer alginate-based scaffolds improve MSC invasion into the host tissue and significantly enhance tissue matrix production.^111^ Substrate stiffness affects the persistence of stem cells *in vivo*^112^^,^^113^ and their adaptation to the host immune^114^^,^^115^ environments. Finally, the matrix stiffness has been shown to affect the morphogenesis of stem cell spheroids and organoids.^116^

The stiffness of the implant drastically changes the original mechanical environment of the tissues adjacent to the implantation site, leading to varied biological responses. For example, remodeling of the peri-implant bone around a stiffness-optimized Ti–35Nb–2Ta–3Zr alloy implant was significantly enhanced compared to Ti–6Al–4V alloy implants under similar loading conditions.^117^ Bone scaffolds (3D-printed) with rationally designed stiffness have demonstrated the ability to promote bone regeneration and ingrowth compared to scaffolds with an arbitrary design of stiffness.^118^^,^^119^ In addition, adjusting the mechanical stress at the muscle injury site using a biomaterial with suitable stiffness can modulate the immune response and reduce fibrosis in the injured muscle.^120^ Myocardial injection of biomaterials into the infarcted and border zones to improve the mechanical properties of the infarcted myocardium is a promising strategy for reducing pathological remodeling after myocardial infarction,^121^^,^^122^ whose therapeutic effect is highly dependent on the stiffness of the injected material.^123^^,^^124^ The influence of substrate stiffness on cell behavior has been discussed extensively in several recent reviews.^77^, ^78^, ^79^, ^80^

#### 3.1.2 Strain-stiffening behavior

Native ECM and many natural biomaterials exhibit strain-stiffening behavior (i.e., stiffness is not constant, but increases with strain) when subjected to large deformations.^125^, ^126^, ^127^ A strain stiffening of a polyisocyanopeptide hydrogel behaviors is displayed in Fig. 6 (b). The stress–strain curve of the hydrogel is in a stress ramp. K’ is its modulus of elasticity.^128^

In fact, many soft tissues also exhibit nonlinear elasticity owing to strain stiffening, which becomes difficult to extend as they are deformed. At the tissue level, the strain-stiffening behavior simultaneously enables tissue deformation under physiological mechanical loads and prevents tissue damage under large loads. For example, in blood vessel walls, distensibility at low strains accommodates pulsatile blood flow, whereas increased stiffness at high strains provides mechanical stability to prevent vessel rupture.^129^

By mimicking the non-linear mechanical behavior of soft tissues, biomaterials with strain-stiffening behavior can better reconstruct the tissue mechanical environment. Importantly, recent findings imply that the strain-stiffening property of gels influences the expression of KCa3.1 potassium channel by mediating cytoskeletal stress fiber formation and affects the functional response of liver carcinoma cells.^130^ Therefore, biomaterials with strain-stiffening behavior bear considerable potential for tissue repair by reconstructing the tissue mechanical environment and directing cellular behavior. For example, hydrogels with strain-stiffening properties facilitate wound repair.^131^^,^^132^

### 3.2 Time-dependent mechanical behavior

Native tissues experience a wide range of loading rates and frequencies, from daily physiological loading to high-rate impact injuries. The time-dependent (or frequency-dependent) mechanical behavior of tissues arising from their intrinsic viscoelasticity and fluid flow-induced poroelasticity is critical to their adaptation to a complicated mechanical environment.^133^ Viscoelastic processes are often associated with stretching and sliding of collagen fibrils (and other macromolecules) within the ECM, whereas poroelastic processes involve local matrix compression and fluid flow. Tissue poroelasticity strongly depends on the experimental length scale, whereas viscoelasticity is independent of the length scale. These two distinct mechanisms are crucial for the physiological functions of connective tissues, such as load bearing, energy dissipation, and storage in cartilage; protection, regulation, and sensing in the skin; and energy dissipation and force transmission in tendons. The time-dependent mechanical properties of soft tissues are increasingly recognized to be linked to several pathological processes. Recently, the effects of viscoelasticity, poroelasticity, and viscosity of biomaterials on cell and tissue responses have been increasingly demonstrated.^35^^,^^134^

#### 3.2.1 Viscoelasticity

Natural tissues and ECM components behave more like viscoelastic or viscoplastic materials, which exhibit prominent stress relaxation behaviors and may undergo permanent deformation (viscoplastic materials) in response to loads.^34^^,^^35^^,^^135^ Viscoelasticity models examine how materials exhibit both elastic and viscous behaviors by analyzing how stress and strain are distributed within the material and how they interact with external forces. Initial attempts to model viscous elastic effects commonly employ springs and dashpots. The Maxwell and Kelvin models are fundamental models used to describe viscoelastic materials. Typical constitutive models for viscoelastic creep and stress relaxation under an instantaneous and constant applied stress (*σ*_*0*_) and strain (*ε*_*0*_) include Spring element, Dashpot element, Maxwell model, and Kelvin/Voigt model. A schematic presentation of common viscoelastic models is displayed in Fig. 6(c),^136^ where *t* represents time, *E* denotes the elastic modulus of spring elements, *η* signifies the viscosity of dashpot elements, and *τ* denotes the relaxation time.

The Maxwell model combines elements of elasticity and viscosity in series, where the total strain experienced by the material is the cumulative effect of both its elastic and viscous strains, corresponding to the following constitutive equations:

$$\dot{\varepsilon }=\frac{\sigma }{\mu }+\frac{\dot{\sigma }}{E}$$

where $\dot{\varepsilon }$ is the strain rate, that is, the derivative of the strain ε with respect to time; μ is the viscosity coefficient of the viscous element; E is the modulus of elasticity of the elastic element; and ε and $\dot{\varepsilon }$ are the stress and stress rate, respectively.

The Kelvin model corresponds to a parallel connection of elastic and viscous elements with the following constitutive equation:

$$\sigma =E\varepsilon +\mu \dot{\varepsilon }$$

The viscoelastic behavior of a material is usually measured using a rheology test and is described by the following equations:

$$G^{*}\left(\omega \right)=G_{e}+iG_{N}^{0}\tau_{0}\omega$$

$$G^{\prime }=G_{e},G^{\prime \prime }=G_{N}^{0}\tau_{0}\omega$$

where $G^{*}$ is complex modulus, ω is oscillation frequency, $G_{N}^{0}$ is the plateau modulus, $G_{e}$ is equilibrium modulus, and $\tau_{0}$ is relaxation time. Stress relaxation and creep tests were also widely used to evaluate the viscoelastic properties of a material.^35^^,^^134^ There are also many other sophisticated viscoelastic models to describe behaviors of biomaterials. For example, a two-layer structural model for healthy young arterial walls was proposed to describe the viscoelastic behaviors.^137^ An orthotropic viscoelastic model was proposed for describing the passive mechanical property of myocardium^138^*.* A novel fractional anisotropic nonlinear viscoelastic model has been suggested to capture observed phenomena in the heart.^57^ This model marks the first instance of a nonlinear anisotropic viscoelastic model for human myocardium that has been shown to accurately match the biomechanical behavior of myocardial tissue and provide credible predictions of tissue response.^139^ A recent study has investigated the use of multiscale mechanical markers to monitor disease progression and treatment. The research revealed a consistent two-stage power-law rheological pattern across various time scales in healthy livers, fibrotic livers, and fibrotic livers treated with mesenchymal stem cells.^140^

Recently, viscoelastic and viscoplastic hydrogels containing dynamic network bonds have attracted considerable attention for tissue repair applications.^126^^,^^127^ The debonding and rebonding processes allow the polymer matrix to flow under the applied stress. Therefore, viscoelastic and viscoplastic polymers can respond to tiny mechanical changes at the cell–material interface to make rearrangements in the polymer network, which could create a self-adaptive mechanical microenvironment facilitating complex cellular functions.^126^^,^^127^ Many cellular behaviors, including focal adhesion formation, cell spreading, maintenance or differentiation of stem cells, cell migration, and ECM synthesis, have been found to be regulated by the matrix viscoelasticity and viscoplasticity rather than by the initial elastic modulus (Table 1).^34^^,^^35^^,^^45^^,^^134^^,^^141^, ^142^, ^143^, ^144^ Recent studies have indicated that substrate viscoelasticity influences immune responses.^145^, ^146^, ^147^ These findings provide a basis for the application of viscoelastic and viscoplastic biomaterials as cell delivery vesicles in tissue repair,^148^ 3D cell/organoid culture platform,^149^^,^^150^ and TE scaffolds for guided tissue regeneration.^151^^,^^152^

The viscoelasticity of delivery vesicles affects cell survival during cell delivery with injection approaches by modulating the shear stress on the cells during delivery by injection. For example, injectable viscoelastic hydrogels with stress dissipation capabilities have been developed for cell transplantation to provide mechanical protection to cells during injection and improve cell viability and retention at the transplantation site post-injection.^153^^,^^154^ In addition, the viscoelasticity of delivery vesicles can regulate cell migration^155^ and differentiation^156^, ^157^, ^158^ and improve the efficacy of cell therapy. Recently, viscoelastic hydrogels have been used to guide the formation of mature organoids,^150^^,^^159^, ^160^, ^161^ which are better representatives of native tissue than cells. Thus, injectable viscoelastic hydrogels can be used for organoid therapy and have great potential in regenerative medicine.^162^

Viscoelastic biomaterials can regulate response of host cells during the repair of tissues, such as nerve,^163^^,^^164^ skeletal muscle,^165^ myocardium,^166^ skin 167,168, bone,^169^ osteochondral tissue,^14^ and intervertebral disc^11^ injuries. Viscoelastic hydrogels are preferred over their elastic counterparts for tissue repair because they direct appropriate cell differentiation^14^^,^^164^^,^^169^ and enable better cell infiltration and tissue integration.^170^

Viscoelastic properties of biomaterials are important for reconstructing the dynamic mechanical environment of load-bearing tissues. For example, when OA occurs, the viscoelastic property of the synovial fluid in joints changes such that its efficacy as a lubricating, shock-absorbing, and filtering agent is diminished,^171^ causing an aberrant mechanoenvironment in the cartilage. In contrast, biomaterials with appropriate viscoelastic behaviors have demonstrated the ability to restore the lubricating and shock-absorbing properties of synovial fluids.^172^ Our recent study revealed that viscoelasticity plays a critical role in allowing epicardial patches to reconstruct the mechanical environment of the myocardium and facilitate tissue restoration after infarction.^10^ The therapeutic effects might due to the protection of mitochondrial function through mitigating the mechanical challenges.

#### 3.2.2 Poroelasticity

Poroelasticity refers to the mechanics of interactions between fluid flow, stress and solid deformation within a porous medium. Porous materials are solid structures that contain empty spaces or voids. Some biopolymer gels and tissues, such as cartilage, intervertebral disc tissue, are poroelastic materials saturated with fluid that flows relative to a deforming solid matrix. These materials exhibit time-dependent responses to mechanical loads, mainly derived from the solid and fluid phase interactions across multiple length scales. Typical poroelastic behaviors of biopolymer networks are schematically presented in Fig. 6(d). When a fluid is extruded within a polymer, it induces a time-dependent normal force along the axial direction. Compressive deformations altering the volume of porous material will inevitably trigger fluid flow through the network due to the incompressibility of fluids. When deformation occurs rapidly, the system behaves akin to an incompressible material due to the load being predominantly sustained by the incompressibility of the interstitial fluid. Conversely, when deformation happens slowly enough to permit fluid outflow, the system behaves like a sponge-like material devoid of fluid, where the compression of voids notably contributes to the reduction in volume. Upon applying a constant shear stress at t ​= ​0, the subsequent decay of normal stress follows an exponential pattern over time. The characteristic time governing this decay is intricately linked to the pore size/shape/connectedness within the network as well as the friction between fluids and solid matrix (permeability of the porous medium). Notably, the biopolymer gels exhibit significantly smaller characteristic time (seconds to minutes) compared to their synthetic counterparts (hours to days), which means that the characteristic time can be sophisticatedly regulated in a rather wide range. For more details, the reader could be referred to ^173^.

The Biot formulation of the constitutive equations for a porous material filled with a fluid is used to describe these responses. The Biot formulation relies on certain key assumptions, including the presumption of linearity in the relationship between stress $\sigma_{ij}$ and strain $\varepsilon_{ij}$, as well as the notion of reversibility in the deformation process, meaning that no energy is dissipated during a closed loading cycle. The most general form of the isotropic material response is.

$$\varepsilon_{ij}=\frac{\sigma_{ij}}{2G}-\left(\frac{1}{6G}-\frac{1}{9K}\right)\delta_{ij}\sigma_{kk}+\frac{1}{3H^{\prime }}\delta_{ij}p$$

$$\zeta =\frac{\sigma_{kk}}{3HH^{\prime }}+\frac{p}{R^{\prime }}$$

where *K* and *G* are thus identified as the bulk and shear moduli of the drained elastic solid, respectively. The additional constitutive constants *H* ′, *H* ″ and *R* ′ characterize the coupling between the solid and fluid stresses and strains, respectively. For the material parameters, refer to ^174^.

The poroelasticity-enabled time-dependent mechanical responses of tissues are critical to their physiological functions. For example, energy dissipation due to poroelasticity occurs under tension or compression resulting from volume changes caused by water flowing into or out of the network. A recent study revealed that poroelastic behavior, together with viscoelastic behavior, play an important role in the physiological functions of connective tissues when adapting to loads across different frequency regimes.^133^

The poroelastic properties of tissues also play essential roles in regulating the homeostasis and function of tissue-resident cells. First, the deformation of the poroelastic tissue results in the flow of interstitial fluid, generating fluid shear stress that affects tissue cell behavior.^175^ Recently, a poroelastic model that associates the relaxation process with fluid diffusion through a fibrous matrix was built to describe local stresses, strains, and velocities sensed by stem cells for a more accurate prediction of stem cell differentiation.^176^ Second, water flowing into or out of the network of poroelastic tissues causes osmotic changes in the cell microenvironment, which is an important stimulus for cell mechanosignaling.^177^ Third, the transport of nutrients from the periphery to the poroelastic tissues and the proper elimination of waste products from the tissues are necessary for tissue survival, which in turn depends on the poroelastic properties of the tissues. This mechanical regulation of tissue nutrition is an indirect mechanism of mechanotransduction.^178^ Fourth, poroelastic tissues such as hyaline cartilage and nucleus pulposus have high thermodissipative capabilities during cyclic deformations, thus providing a dynamic thermal milieu for the residing cells. A recent study revealed that the self-heating property of poroelastic materials is important for cells to effectively perceive and translate the applied mechanical loading,^179^ which could be viewed as another indirect mechanotransduction mechanism.

#### 3.2.3 Viscosity

Viscous liquids “flow” in response to stress, with strain increasing linearly (and irreversibly) with time when a constant force is applied. The viscosity is a key property of viscous liquids. A schematic diagram of Newton's law of viscosity can be found in Fig. 6(e). The shear stress can be expressed as *τ* ​= ​*μ* (d*u*/d*y*), where *τ* is the shear stress, *μ* is the kinematic viscosity of the liquid, and d*u*/d*y* is the rate of shear deformation.

Various models have been proposed to describe the viscosities of biomaterials.^180^ The generalized form of Newton's law of viscosity can be written as

$$\tau =\mu \left[\nabla v+{\left(\nabla v\right)}^{*}\right]-\left(\frac{2}{3}\mu -\kappa \right)\left(\nabla \cdot v\right)\delta$$

where τ is the viscous stress tensor, flow velocity is denoted as v, the dagger ∗ denotes the transpose, δ is the unit tensor, μ and κ denote the shear and bulk viscosities, respectively.

The influence of matrix viscosity on cellular behavior has also been revealed (Table 1).^134^^,^^181^, ^182^, ^183^, ^184^ For example, substrate viscosity regulates the collective movement of epithelial cells.^181^ Viscosity-dependence of cell spreading and spheroid formation was observed on poly(ε-caprolactone)-based substrate.^183^ More viscous surfaces increase the amount of mechanosensitive YAP in the nucleus and enhance myoblast differentiation.^184^

The effect of viscosity on cell behavior in 3D cultures has been investigated by embedding a viscous gelatin solution in chemically crosslinked gelatin hydrogels.^185^, ^186^, ^187^ Chondrocytes have a more spread morphology, higher proliferation, lower ECM production, and decreased expression of collagen type II and aggrecan in a low-viscosity gelatin solution than in a high-viscosity solution, suggesting that high viscosity is more beneficial for the maintenance of the chondrocyte phenotype, whereas low viscosity is more beneficial for cell expansion.^185^, ^186^, ^187^ Using a similar system, the authors revealed that a gelatin solution promoted the proliferation of MSCs, but its promotive effect decreased with an increase in viscosity.^186^ Importantly, the results showed that high viscosity is beneficial for the osteogenic differentiation of MSCs, whereas low viscosity is beneficial for adipogenic differentiation.^186^ High-viscosity gelatin solution is also beneficial for chondrogenic differentiation of hMSCs under the synergistic stimulation of chondrogenic induction factors.^187^ Recently, a high-viscosity buffered red blood cell storage solution was demonstrated to mitigate many aspects of red blood cell storage lesions and the inflammatory response to resuscitation after hemorrhage.^188^

These studies suggest that viscosity should be considered as one of the key mechanical cues affecting cell–material interactions and is an important factor in engineering materials that control cell behavior.

### 3.3 Programming mechanical properties of biomaterials to control cell/tissue responses

Biomaterials that exhibit stimuli-responsive changes in mechanical characteristics can temporally exert dynamic mechanical stimuli to adherent cells or restore tissue mechanical environment adaptively,^80^^,^^191^ offering an opportunity to emulate the dynamics of physiological mechanical environment for stimulating tissue repair.^126^^,^^127^ Biomaterials that exhibit stimuli-responsive changes in their mechanical characteristics can temporally exert dynamic mechanical stimuli on adherent cells or adaptively restore the tissue mechanical environment,^80^^,^^191^ offering an opportunity to emulate the dynamics of the physiological mechanical environment to stimulate tissue repair. This type of biomaterial has been explored as a novel platform for steering cell behavior,^190^^,^^192^, ^193^, ^194^, ^195^ revealing cellular mechanobiological responses that are not captured by static biomaterial platforms. First, the dynamic substrate stiffness reversibly controls cell morphology.^196^ Second, the dynamic substrate affects the commitment and differentiation of stem cells and their spheroids.^197^^,^^198^ Third, the cell phenotype may change with alterations in substrate stiffness.^199^ Thus, biomaterials offer a unique opportunity for tissue engineering applications, where in situ dynamic alterations of matrix mechanical properties can be programmed to adaptively emulate the mechanical environment of the native extracellular matrix.^200^ At load-bearing sites, this type of biomaterial can consistently restore the dynamic tissue mechanical environment in adaptation to the tissue healing process, thereby optimizing the dynamic mechanical signals transmitted to biomaterial- or tissue-resident cells.^201^^,^^202^

Biomaterials with stimuli-responsive alternating geometries have also been developed.^203^^,^^204^ Under the actuation of external stimuli, the geometries of these materials change in a programmed manner, actively exerting mechanical forces on the adherent cells and contacting tissues. Such biomaterials provide opportunities to exert mechanical stimuli on tissues/cells that cannot be realized by physiological activities. Efforts have been made to elucidate the effects of biomaterial-programmed dynamic mechanical stimuli on cells.^205^, ^206^, ^207^ For example, the remote stimulation of an ultrasound-responsive Janus scaffold resulted in mechanical nanovibration that transmitted dynamic mechanical signals to adherent hBMSCs, which enhanced cell proliferation, matrix deposition, and osteogenic differentiation via the formation and activation of voltage-gated calcium ion channels.^207^ Acoustic radiation force was successfully delivered to cells in gelatin methacryloyl hydrogels using acoustic waves, which induced cell patterning.^208^ These findings have inspired researchers to develop advanced materials and devices that deliver dynamic mechanical stimuli to cells. This type of biomaterial is capable of exerting mechanical stimuli on the surrounding tissue in a controlled and programmed manner to enhance tissue regeneration. For example, alginate-based hydrogels impregnated with iron oxide nanoparticles enhanced healing in a mouse muscle injury model by applying cyclic mechanical loading to the tibialis anterior under an oscillating magnetic field.^209^ Similarly, periodic axial muscle stretching applied by an injectable, biodegradable magnetic hydrogel system was superior to massage-like compression in maintaining muscle mass and structure in an animal model, suggesting pathways for combating muscle disuse atrophy.^15^ A mechanically active gel-elastomer-nitinol tissue adhesive (MAGENTA) was developed to generate and deliver muscle contraction-mimicking stimulation to target tissues with programmed strength and frequency,^210^ which activated mechanosensing pathways involving yes-associated protein and myocardin-related transcription factor A, and increases the rate of muscle protein synthesis. Disused muscles treated with MAGENTA exhibit greater size and weight and generate higher forces than untreated muscles, demonstrating their potential for atrophy mitigation.^210^ In addition, dressings that can actively contract wounds in response to skin temperature accelerate skin wound healing.^16^ Similarly, injectable self-healing hydrogels with thermoresponsive self-contraction and tissue adhesion properties have been shown to assist in wound closure by actively contracting wounds.^211^ These studies demonstrate the great promise of programming dynamic mechanical stimuli from biomaterials for tissue repair and regeneration. However, the mechanobiological mechanisms behind the therapeutic effects remains to be revealed.^4^

## 4 Theoretical tools for deciphering mechanical interactions and designing optimal mechanics and geometry

The development of mechanobiomaterials relies on better prediction of the mechanical interaction between the biomaterial and host tissue/cell to characterize the mechanical cues perceived by cells. Numerical simulation approaches have demonstrated great promise for deepening the mechanistic understanding and guiding the rational design of mechanobiomaterials.

**Fig. 7:**
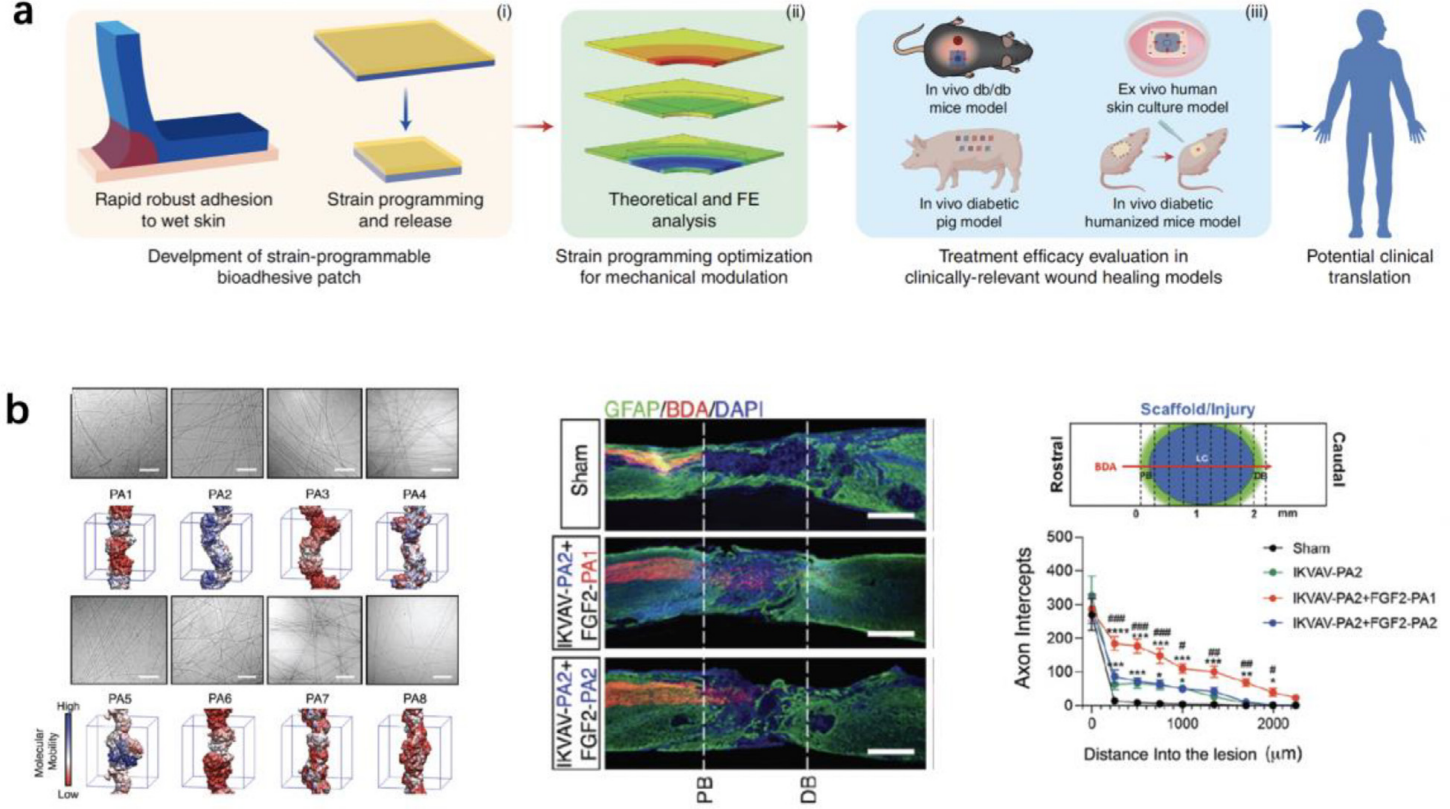
Application of simulation approaches in the development of mechanobiomaterials. (a) With the aid of FEA, a strain-programmed patch was developed, which could mechanically contract diabetic wounds in a programmable manner to enhance the healing of diabetic wounds.^217^ (b) Guided by coarse-grained MD simulations, peptide amphiphiles (PAs) based assemblies with high internal dynamics was developed for spinal cord injury repair.^163^

Numerical simulation approaches have been widely used to determine the mechanical properties of implants. Finite element analysis (FEA) is typically used to investigate material/tissue interactions to optimize the mechanical and structural properties of medical devices, including bone screws,^212^ tissue engineering scaffolds,^213^^,^^214^ stents,^215^^,^^216^ and epicardial patches.^10^ For example, a strain-programmed patch that implemented a hydration-based shape-memory mechanism was developed to mechanically contract diabetic wounds in a programmable manner (Fig. 7 a).^217^ The optimal mechanical and geometric parameters of the patch were determined using FEA. The patch enhanced the healing of diabetic wounds by promoting faster re-epithelialization and angiogenesis as well as the enrichment of fibroblast populations with a pro-regenerative phenotype in various animal models.^217^ FEA has also been used to explain or predict the biological responses by coupling established mechanobiological mechanisms with tissue or cell stress/strain.^2^, ^218^

FEA aims to employ and solve relevant partial differential equations to describe physical phenomena within materials and structures on a continuum scale (usually on a millimeter scale for biological systems). As such, it does not model the interactions between individual molecules. In contrast, molecular dynamics (MD) is used for microscopic simulations of interatomic and intermolecular interactions. MD simulations have been used to study material/cell interactions at the subcellular or molecular level and the dynamics of hydrogel networks.^163^^,^^219^^,^^220^ For example, in the development of scaffolds for spinal cord injury repair, the internal dynamics of various peptide amphiphilic (PAs)-based assemblies were compared using coarse-grained MD simulations (Fig. 7 b).^163^ In vitro and *in vivo* studies have revealed that the degree of internal dynamics is critical to the bioactivity of scaffolds,^163^ which resulted in notable differences in vascular growth, axonal regeneration, myelination, survival of motor neurons, reduced gliosis, and functional recovery.^163^ Recently, using coarse-grained MD simulations, the receptor-mediated endocytosis of elastic nanoparticles of different sizes and shapes was systematically investigated,^220^ providing a detailed mechanistic understanding of the influence of nanoparticle size, shape, and elasticity on membrane-wrapping efficiency, which serves as a foundation for the rational design of nanoparticle-based drug carriers.

## 5 Rational design of mechanobiomaterials

**Fig. 8:**
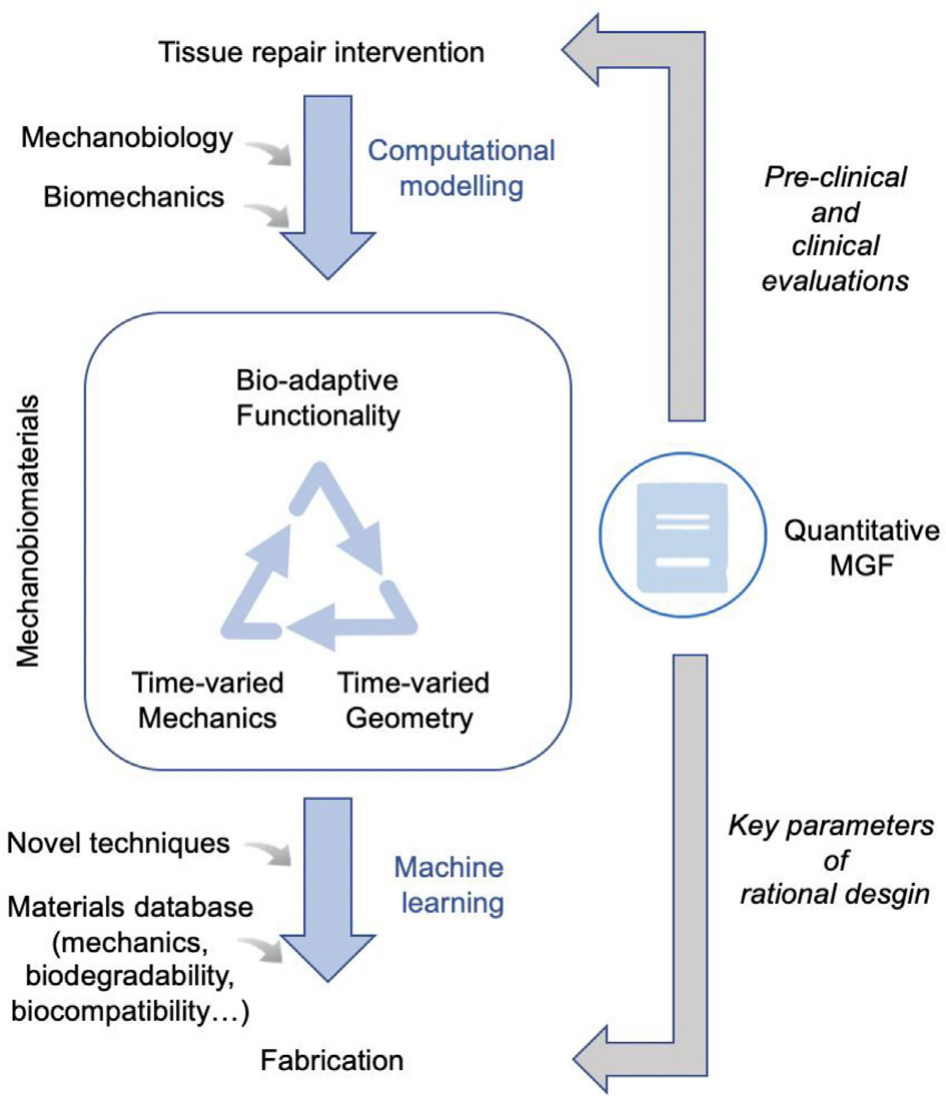
A closed-loop workflow of designing mechanobiomaterials.

A thorough understanding of the mechanobiological effects of materials on cell and tissue functions is the foundation for the rational design of mechanobiomaterials to promote tissue repair and regeneration. One of the basic considerations of mechanobiomaterials is the optimization of the dynamic mechanical interactions between tissues and biomaterials to facilitate tissue repair and regeneration. When exogenous cells are incorporated into biomaterials for cell-based therapy, another basic consideration is the optimization of the mechanical environment of the cells to enable their ability to mediate tissue regeneration. Based on these two considerations, a typical workflow for the inverse design of mechanobiomaterials was proposed (Fig. 8). The rational integration of a material's mechanical properties and geometry/structure to achieve targeted effects on biological functions is important, but also challenging. In particular, physical models and simulation approaches that can decipher the mechanical interactions and mechanisms between materials and biological systems are necessary.

### 5.1 Function-oriented optimization of mechanics and geometry

**Fig. 9:**
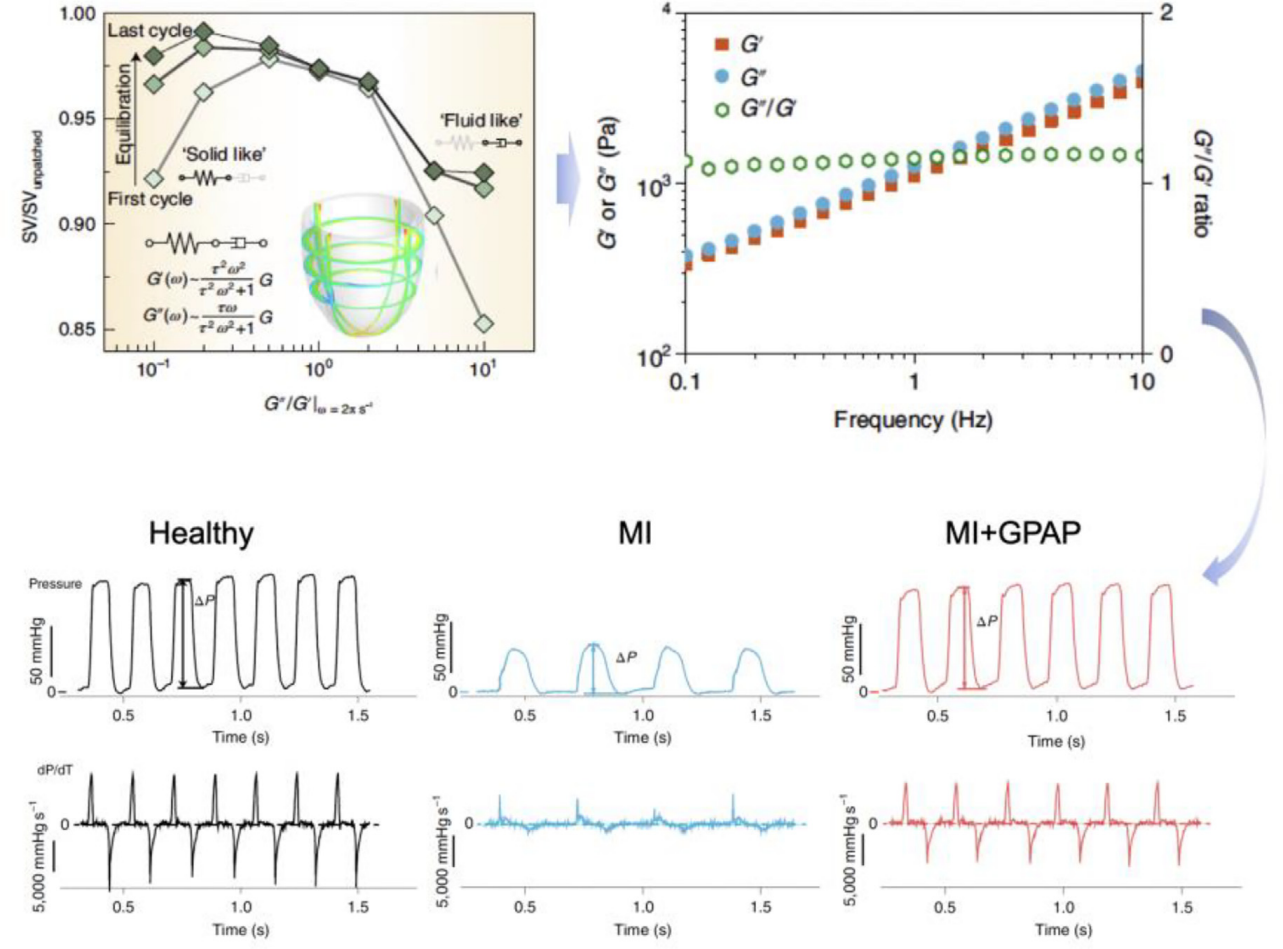
Development of a mechanobiologically optimized epicardial patch using a closed-loop workflow of reverse design.^10^

The design of mechanobiomaterials can sometimes be deemed as the reverse engineering of the material's mechanical properties and geometries, according to the established knowledge of mechanobiology and analysis of multiscale mechanical interactions between biological systems and biomaterials. A typical workflow is as follows:

Computational models allow the simulation of biomechanical processes under normal and pathological conditions based on the quantitative analysis of changes in tissue mechanics and geometry after tissue lesions. Biomaterials with different mechanical properties and geometries were included in the computational model to capture the dynamics of the mechanical environment in biological systems and biomaterials. Computational models based on mechanobiology should aim to predict how biomaterials affect the mechanobiological processes of tissue or biomaterial-resident cells through mechanical interactions. After assessing the biological impact of biomaterials on target tissues/cells, mechanics-geometry-function (MGF) relationships can be established at multiple scales, and key mechanical/geometric parameters can be identified and optimized to maximize the therapeutic efficacy of biomaterials. Then, the key mechanical/geometrical parameters and their optimal values can be used to design biomaterials and their fabrication processes with the aid of machine learning and/or other novel approaches based on material databases (e.g., mechanical properties, biodegradability, and biocompatibility). Finally, the designed biomaterials were prepared and investigated using material characterization and biological and preclinical/clinical evaluations to verify the success of the design. The closed-loop workflow is shown in Fig. 8. Our research team used this closed-loop workflow and developed a viscoelastic epicardial patch with an optimized shear modulus and loss factor to restore the dynamic mechanical environment of the infarcted myocardium for MI treatment (Fig. 9).^10^ In this study, the mechanical properties and geometry of the epicardial patch were optimized by deciphering the mechanical interactions between the biomaterial and the myocardium through computational modelling. The optimal epicardial patch can restore the systolic function of left ventricle to nearly normal levels.^10^

Such a reverse design strategy inverts the conventional “trial and error” paradigm of biomaterial development by starting with a set of desirable functionalities and searching for the optimal designs and fabrication routes. The essence of mechanobiomaterials is to achieve the reverse design of biomaterials through the computationally aided optimization of mechanics and geometry. This approach not only assists in the design of biomaterials with improved functionalities, but also represents a new paradigm compared to current practices in biomaterial development, where the mechanical properties and geometry of the biomaterials are often adjusted according to large quantities of tests and data analysis. A reverse-design approach for mechanobiomaterials could help reduce technological and experimental efforts to produce biomedical devices for regenerative medicine.

### 5.2 Integration and synergy of material mechanics and biology

Native tissues exhibit a combination of spatiotemporally varying mechanical characteristics at multiple length scales to adapt to their dynamic mechanical environments, maintain physiological functions, and deliver appropriate mechanical stimuli to tissue cells. Inspired by native tissues, the integration of mechanical characteristics with different time dependences at different length scales in a spatiotemporally controlled manner is critical to the design of mechanobiomaterials that are capable of simultaneously re-constructing the mechanical environment for both tissue- and biomaterial-resident cells.

For re-constructing the mechanical environment of constantly deformed tissues, both the stiffness and stress-relaxation behaviors of biomaterials should be tailored to accommodate the time-varying tissue deformation. A prominent example is our viscoelastic epicardial patch with optimized stiffness and stress-relaxation behavior to restore the dynamic mechanical environment of the infarcted myocardium for MI treatment;^10^ which was identified to effectively prevent adverse left ventricular remodeling, whereas the stress-relaxation behavior was designed to maintain the pumping function of left ventricle. Recently, we developed a glycerol-crosslinked PVA gel (GPG) for the treatment of IVDD,^11^ which has a nucleus pulposus (NP)-matched shear modulus to restore the mechanical environment of IVD tissues under static loading while possessing a prominent energy dissipation capability to accommodate the dynamic loading environment. The injection of GPG efficiently restored IVD height in a needle puncture model and prevented IVD pathological remodeling.^11^

**Fig. 10:**
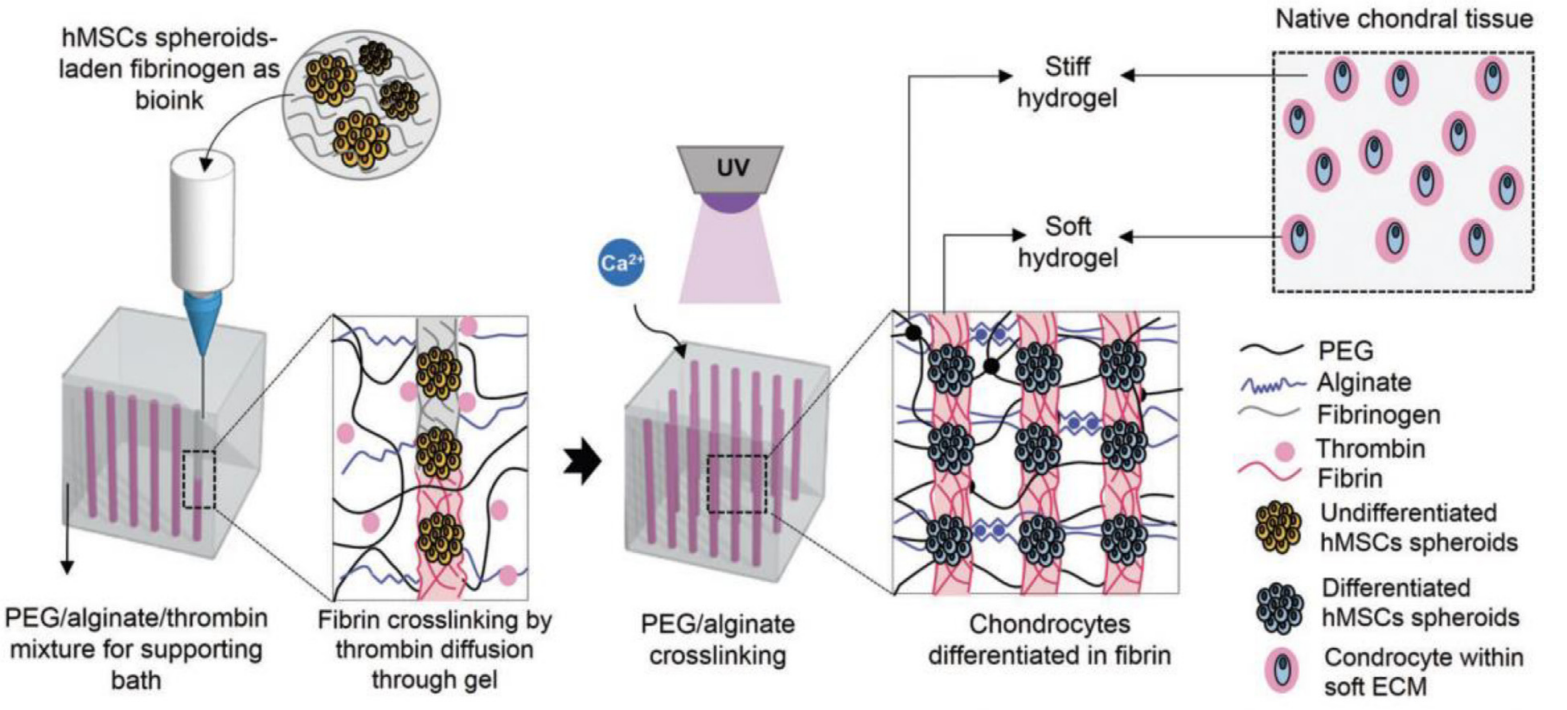
Schematic of a 3D bioprinting approach for engineering cartilage-like mechanically hierarchical constructs with a hard biomaterial to mimic the macro-mechanical properties of native cartilage, and a soft biomaterial to create a chondrogenic microenvironment.^222^

Natural ECM in load-bearing tissues is both macroscopically stable to withstand repeated biomechanical challenges and microscopically dynamic to allow for cellular activities.^173^^,^^221^ Recapitulating the mechanical characteristics of the ECM in synthetic hydrogels is critical for many biomedical applications. Recently, cartilage-like tissue was fabricated by 3D printing using two biomaterials with different mechanical properties: a hard biomaterial (with a compressive modulus on the order of MPa) to achieve the macro-mechanical properties of cartilage, and a soft biomaterial (with a compressive modulus on the order of kPa) to create a chondrogenic micromechanical environment (Fig. 10).^222^ The hierarchical mechanical design ensured appropriate mechanical stimuli for the hMSC spheroids in the scaffold, resulting in high viability and chondrogenic-like behavior.^222^ It is worth noting that cells in scaffold would also affect the macro-mechanical characteristics of scaffolds under external load via the “tensional homeostasis” mechanism,^59^ which should be taken into consideration for rational design of cell-laden mechanobiomaterials for repair of load-bearing tissues.

Spatiotemporal control of mechanical properties is also critical for the precisely reconstructing cell/tissue mechanical environment. An example is the development of a bilayer osteochondral scaffold with a 3D-printed Ti alloy subchondral bone compartment and a freeze-dried poly-lactic-co-glycolic acid (PLGA) reinforced collagen sponge as cartilage compartment was developed.^223^ The mechanical support provided by the 3D-printed Ti alloy layer facilitates long-term regeneration of cartilage by accelerating osteochondral formation and its integration with the adjacent host tissue. Recently, osteochondral scaffolds with spatially heterogeneous stiffness demonstrated the stiffness-specific induction of chondrogenic and osteogenic differentiation of stem cells for cartilage and bone regeneration, respectively.^189^ Most recently, the multidirectional stiffness design of artificial IVD demonstrated the ability to simulate the physiological kinematic behaviors of native IVD, bearing great potential in reconstructing the mechanical environment of adjacent tissues.^224^

### 5.3 Structural and geometrical optimization

By resorting to a microgeometry-driven approach, the design domain of biomaterials is becoming much wider, which also enables a more accurate modulation of tissue/cell–material mechanical interactions. By tailoring the geometrical parameters of the unit cells as basic building blocks, desirable mechanical properties can be obtained,^225^^,^^226^ such as tunable Poisson's ratio,^227^ chirality,^228^ and high specific stiffness.^229^ Certain tissues in the human body, such as tendons, skin, annulus fibrosus disks, arteries, and cancellous bone, exhibit auxetic characteristics when subjected to tensile forces. Auxetic materials can serve as scaffolds in tissue engineering. These materials not only demonstrate unique mechanical attributes but can also enhance cell proliferation when subjected to external loads.^230^

Numerous studies have highlighted the potential of auxetic materials for tissue engineering and biomedical devices. In particular, auxetic materials have great potential for the design and fabrication of mechanobiomaterials^231^ because their effective mechanical properties can be tuned by modifying the underlying microstructures, thereby reproducing the mechanical behaviors of skin tissue, arteries, tendons, IVD, and cancellous bone.^230^^,^^232^^,^^233^ Numerous auxetic structures have been successfully fabricated and investigated based on various microstructure design motifs including “re-entrant”,^234^ “chiral”,^235^ and “rotating”^231^ types of unit cell.

**Fig. 11:**
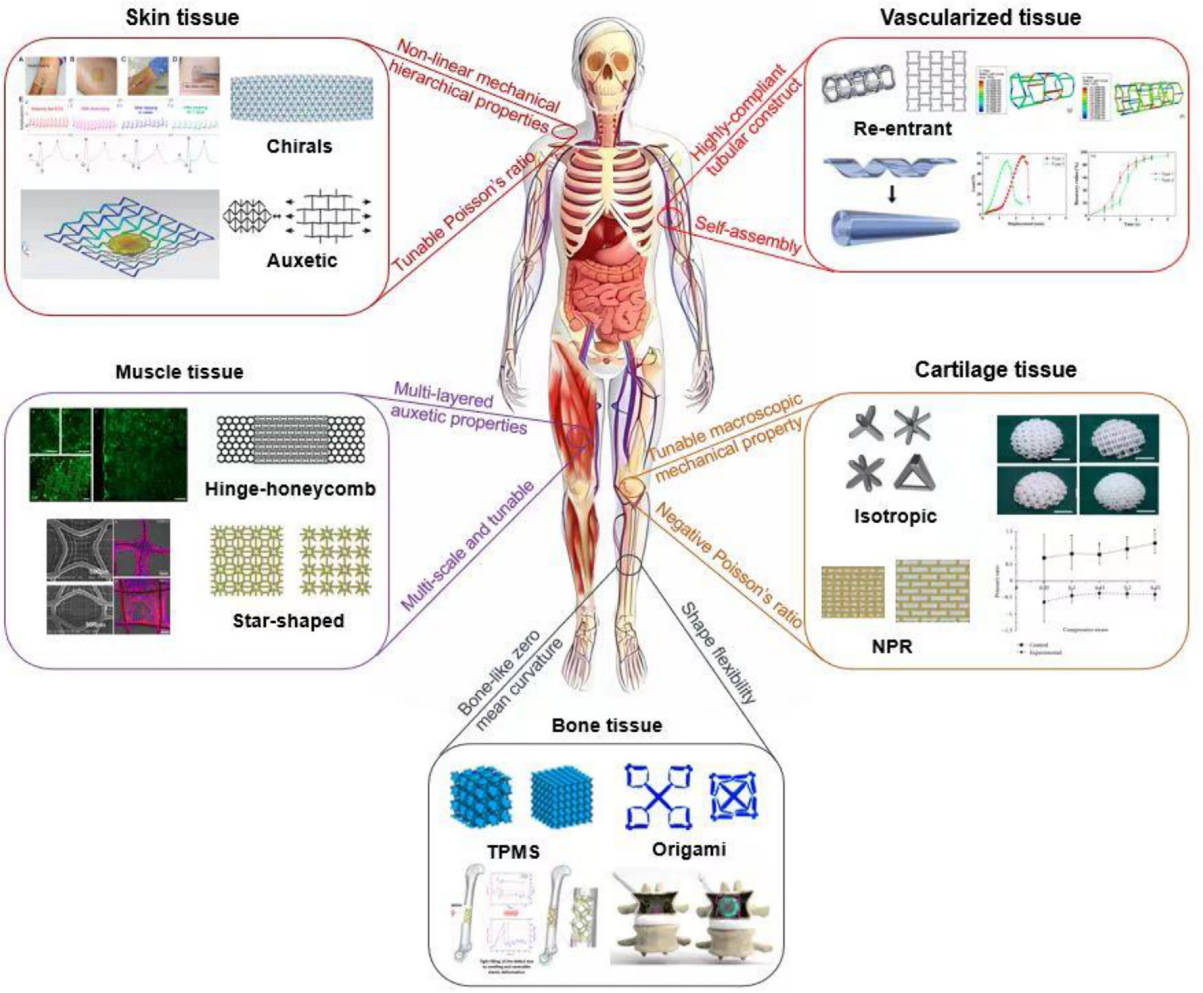
Various auxetic materials for biomedical applications.^239^^,^^246^, ^247^, ^248^, ^249^, ^251^, ^252^, ^253^, ^254^, ^255^

Auxetic materials have also gained substantial attention for biomedical device applications (Fig. 11). In particular, artificial auxetic polyurethane IVDs possess a remarkable ability to bend and twist, potentially offering superior biomechanical performance compared with conventional disc replacement options. Because of their anisotropic negative Poisson's ratio, these discs effectively prevent the occurrence of bulges that may harm nearby nerve endings.^233^ Importantly, these discs closely replicated the behavior exhibited by natural lumbar IVDs.^233^ An auxetic pedicle screw was created using a re-entrant unit cell structure,^236^ achieving outstanding screw-bone fixation performance in spinal surgery. It was found that implant-bone contact and implant longevity were improved by using auxetic-based hybrid implants when pressing onto the bones from both the medial and lateral sides.^237^ An innovative auxetic coronary stent enables the stent maintains a specific lumen volume by simultaneously expanding in two directions when the balloon is inflated.^238^ Analysis of the stent diameter and length before and after tension revealed that the auxetic coronary stent exhibited radial and longitudinal expansion. This expandability is advantageous because it allows the stents to adapt to different vessel dimensions. Other applications include an auxetic material patch for the treatment of myocardial infarction,^239^ where the unique property of having a negative Poisson's ratio allows the patch to effectively adapt to the intricate mechanical requirements of the heart, as well as auxetic materials used in the neck^240^ and ankle braces.^241^

The presence of auxetic properties in several biological tissues has sparked interest in the use of auxetic materials for tissue engineering.^230^ Studies have indicated that auxetic structure-based scaffolds establish a biophysical environment that influences the organization of actin and vascular differentiation of pluripotent stem cells.^242^^,^^243^ Several studies have examined the effects of loading cells within auxetic scaffolds in a dynamic loading environment mimicking physiological conditions.^244^^,^^245^

Biomaterials with programmable mechanical properties and structures/geometries can actively exert mechanical stimuli on tissues and cells. Various modelling approaches have shown great promise for describing the mechanical interactions between materials and biological systems at the tissue, cellular, and subcellular levels. In the proposed closed-loop reverse design of mechanobiomaterials, mathematical/physical modeling and simulation approaches play a critical role in establishing MGF relationships at multiple scales to identify and optimize the key mechanical/geometrical parameters of biomaterials with the aim of maximizing their therapeutic efficacy. Data-driven machine-learning approaches will play increasingly important roles in the design and fabrication of mechanobiomaterials.^250^

## 6 Summary and outlook

Recently, mechanobiology of living tissues has been increasingly incorporated into the design of biomaterials with desirable biological functions. Programmed mechanical interactions between biomaterials and host tissues/cells can attenuate inflammatory responses, regulate the activity of regeneration-related cells, and reconstruct physiologically relevant mechanical environments that are crucial for tissue repair and regeneration. Based on an increasing number of studies devoted to the development of mechanobiologically optimized materials with enhanced tissue repair and regeneration capabilities, a new field of mechanobiomaterials is emerging. This field represents a paradigm shift in biomaterial engineering from passive adaptation to tissue mechanical environment to proactive regulation of the tissue mechanical environment. This article aims to profile this newly emerging field by summarizing the existing theoretical and experimental underpinnings, development, and design tools, as well as selective examples of success in this field.

Despite rapid development, the field of mechanobiomaterials remains at a very early stage and their potential for tissue repair and regeneration is yet to be fully realized. The design of mechanobiomaterials is still limited by a lack of quantitative guidelines for optimizing the mechanical parameters. Although some basic guidelines have been established, as summarized in this review, a systematic framework with qualitative and quantitative understanding of MGF relationships is still lacking. Further understanding of MGF relationships relies on novel biomaterial fabrication techniques, a better description of the mechanical interactions between the implant and host tissues, and clarification of cell response mechanisms to mechanical stimuli. Advances in the following aspects are expected to significantly promote the development of mechanobiomaterials and their translation from bench to bed. (1) The role of material mechanics in regulating the biology of material-resident cells needs to be delineated. (2) The connections between the tissue mechanical environment and tissue cell signaling, transcription factor activation, and the epigenome require further exploration. (3) Synergistic effects of mechanical and other physicochemical cues must be elucidated. Multidisciplinary collaborations across biology, materials science, biomedical science, engineering, and other sister disciplines are indispensable to address these challenges. In addition, data-driven machine learning and artificial intelligence tools can be used to accelerate the progress in this field. Current MGF relationships can be utilized in both the design and fabrication of mechanobiomaterials that start from target functionalities and generate the corresponding design parameters for subsequent fabrication.

Ideally, the mechanical properties of the implant material should dynamically match those of native tissue during the entire healing process. However, achieving dynamic mechanical regulation of matching between the implant and tissue during healing progression remains impossible. Biomaterials that have dynamic mechanics in response to time-varied physiological environment can shed light on developing better mechanobiomaterials that consistently adapt to the local mechanical environment for reconstructing tissue homoeostasis or promoting tissue regeneration. Therefore, advances in this type of biomaterials are expected to provide essential technological basis for fast development of mechanobiomaterials. Generally, biomaterials with dynamic mechanics could be achieved by stimuli-responsive property design and self-regulation ability design.

Despite many outstanding challenges, the emerging field of mechanobiomaterials is creating new strategic avenues for tissue regeneration and repair. Further understanding and application of MGF relationships would not only allow the customization of next-generation functional biomaterials, but also provide important guidelines for the development of novel biomedical devices and therapeutics. Joint efforts by materials engineers, mechanical scientists, biologists, and clinicians are required to advance this field.

## CRediT authorship contribution statement

**Xiao Lin:** Funding acquisition, Writing – original draft. **Hua Yang:** Writing – original draft. **Yi Xia:** Writing – original draft. **Kang Wu:** Writing – original draft. **Fengcheng Chu:** Writing – original draft. **Huan Zhou:** Writing – original draft, Funding acquisition. **Huajian Gao:** Supervision, Writing – review & editing. **Lei Yang:** Funding acquisition, Supervision, Writing – review & editing.

## Declaration of competing interest

The authors declare that they have no known competing financial interests or personal relationships that could have appeared to influence the work reported in this paper.
